# Treatment of colorectal peritoneal metastases with oxaliplatin induces biomarkers predicting response to immune checkpoint blockade

**DOI:** 10.1016/j.tranon.2025.102464

**Published:** 2025-07-01

**Authors:** Alexander Constantinides, Nico Lansu, Peter Mosen, Paulien Rauwerdink, Esther Strating, Franziska Völlmy, Maaike Nederend, Jeanette H.W. Leusen, Koen Rovers, Emma Wassenaar, Robin Lurvink, Maarten Altelaar, Simon Nienhuijs, Rene Wiezer, Inne H.M. Borel Rinkes, Djamila Boerma, Geert J.P.L. Kops, Ignace de Hingh, Onno Kranenburg

**Affiliations:** a Laboratory Translational Oncology, Division Imaging and Cancer, University Medical Center Utrecht, Utrecht, the Netherlands; b Department of Surgical Oncology, Division Imaging and Cancer, University Medical Center Utrecht, Utrecht, the Netherlands; c Hubrecht Institute, Royal Netherlands Academy of Arts and Sciences (KNAW), and University Medical Center Utrecht, Utrecht, the Netherlands; d Oncode Institute, Utrecht, the Netherlands; e Biomolecular Mass Spectrometry and Proteomics, Bijvoet Center for Biomolecular Research, Utrecht Institute for Pharmaceutical Sciences, Utrecht University and Netherlands Proteomics Centre, the Netherlands; f Department of Surgery, Antonius Hospital, Nieuwegein, the Netherlands; g Center for Translational Immunology, University Medical Center Utrecht, Utrecht, the Netherlands; h Department of Surgery, Catharina Hospital, Eindhoven, the Netherlands; i Department of Epidemiology, GROW-School for Oncology and Developmental Biology, Maastricht University, Maastricht, the Netherlands; j Utrecht Platform for Organoid Technology, Utrecht University, Utrecht, the Netherlands

**Keywords:** CRC, PIPAC, Peritoneal metastasis, Karyotype, Heterogeneity, Colorectal, Oxaliplatin, Tertiary lymphoid structure, Reverse translation

## Abstract

**Background:**

Colorectal cancer (CRC) patients with inoperable peritoneal metastases (PM) have a dismal prognosis with limited treatment options. Local treatment of CRC-PM with oxaliplatin is commonly applied, but biomarkers steering patient selection, or informing potentially effective combination therapies are lacking. A novel potentially effective treatment strategy is Pressurized IntraPeritoneal Aerosol Chemotherapy (PIPAC) in which CRC-PM are exposed to cyclic treatment with high concentrations of locally applied oxaliplatin. However, it is unclear whether and how CRC-PM respond to PIPAC.

**Methods:**

Here, we generated a biobank from 20 patients receiving PIPAC with oxaliplatin for CRC-PM. The biobank contains biopsies from 3 PM per patient, repeatedly sampled prior to each treatment cycle, and ascites. Anti-tumor effects were analyzed by shallow single-cell karyotype sequencing (sc-karyoSeq). RNA-sequencing and proteomics were performed to assess changes in gene and protein expression. Immunohistochemistry was performed to assess treatment-induced changes in tissue histology. Ascites was used to assess immunoglobulin content and reactivity.

**Results:**

PIPAC reduced genomic heterogeneity and aneuploidy scores among PIPAC-surviving tumor cells. Furthermore, PIPAC reduced immunosuppressive signals (hypoxia, interleukin-10, transforming growth factor β), and induced an influx of B and T lymphocytes, which organized into metastasis-associated Tertiary Lymphoid Structures (TLS). TLS are biomarkers predicting response to Immune-Checkpoint Inhibitors (ICIs). The T cells residing in PIPAC-induced TLS expressed high levels of the checkpoints PD-1, TIGIT and EBI3. PIPAC also caused the generation of plasma cells producing tumor-reactive antibodies.

**Conclusion:**

PIPAC shows modest anti-tumor activity and induces immune parameters predicting response to ICIs. Patients with inoperable CRC-PM may therefore benefit from PIPAC in combination with ICIs.

## Highlights

• Colorectal cancer patients with peritoneal metastases have a dismal prognosis.
• The PIPAC procedure allows repeated intraperitoneal delivery of oxaliplatin.
• A tumor tissue biobank was created from patients receiving PIPAC treatment.
• PIPAC induces biomarkers predicting response to immune checkpoint inhibitors.
• Combining PIPAC with immune checkpoint inhibitors may yield clinical benefit.

## Introduction

Patients with irresectable peritoneal metastases from colorectal cancer (CRC) have a poor prognosis and derive little benefit from systemic therapy [1]. Pressurized intraperitoneal aerosol chemotherapy with oxaliplatin (PIPAC) is currently being evaluated as an alternative treatment strategy in this patient category [2]. The procedure itself is considered feasible and safe and is well tolerated [2,3]. In studies reporting the application of PIPAC, patients often received concomitant systemic therapy, which hampers the interpretation of response data. Therefore, the clinical benefit of PIPAC in CRC patients with peritoneal metastases remains to be established [2,3]. The PRODIGE-7 study recently showed that Hyperthermic Intraperitoneal Chemotherapy (HIPEC), using oxaliplatin as a single drug following resection of peritoneal metastases from CRC combined with systemic therapy, yields no clinical benefit [4]. As a result, routine use of oxaliplatin in the adjuvant treatment of peritoneal metastases following surgery has been challenged, and this has had a worldwide negative impact on application of the procedure [5]. Importantly, the PRODIGE-7 study did not provide novel insights into the mechanisms that may underlie the observed lack of clinical benefit. Therefore, studies aiming to optimize the intra-abdominal treatment of peritoneal metastases from CRC are urgently needed [5]. This requires insight into the biology of peritoneal metastases and how such lesions respond to local treatment with oxaliplatin and other drugs [1].

To address this issue, we performed an extensive analysis of patient-derived tissue and ascites samples from the phase II CRC-PIPAC study [6] in which patients with inoperable peritoneal metastases were exposed to multiple cycles of intraperitoneal oxaliplatin. A unique aspect of the PIPAC procedure is that it provides repeated access to peritoneal metastases in cancer patients as they undergo a laparoscopy prior to intraperitoneal drug treatment. This provides an ideal opportunity to shape ‘reverse translational research’ (i.e. from bedside to bench) [7]. Biopsies from three distinct peritoneal metastases were prospectively collected in every patient prior to each treatment cycle, and ascites whenever present. This yielded a CRC-PIPAC biobank that allowed us to study the longitudinal genetic, cellular, and molecular response of peritoneal metastases to repeated intraperitoneal oxaliplatin exposure without concomitant systemic therapy. We hypothesized that a thorough molecular and cellular analysis of PM receiving intraperitoneal oxaliplatin treatment could lead to the discovery of biomarkers informing future combination treatment strategies with PIPAC. The results demonstrate that PIPAC induces influx of T cells and B cells and the formation of tertiary lymphoid structures (TLS). The T cells express the immune checkpoints PD1 and TIGIT. Therefore, we conclude that the combination of PIPAC with immune checkpoint inhibition may be an effective strategy to combat CRC-PM.

## Methods

For all methods, please see the supplemental information file.

## Results

### Generation of a longitudinal CRC-PIPAC biobank

Twenty patients participating in the CRC-PIPAC study received 1–6 cycles of intra-peritoneal aerosolized oxaliplatin, without concomitant systemic therapy [6]. In total, the biopsy and ascites/flush sample collection was performed during 59 procedures. This effort yielded 169 biopsies, 36 ascites samples and 21 flush samples (Table S1). Biopsy extraction failed in 8 cases (4.5 %). Ascites or flush samples failed to be collected during 3 procedures (5 %). The participating hospitals analyzed the ascites using conventional cytology and malignant cells were detected in 19 of the 57 ascites/flush samples. Tumor organoid cultures were successfully derived from 10 of these samples.

### PIPAC reduces intra-tumor genetic heterogeneity and selects for cells with simpler karyotypes

Although radiological imaging failed to document response in the CRC-PIPAC study, the biological, pathological, cytological, and ascites response rates varied from 50–67 % [6]. In addition, PIPAC caused a significant reduction of the median macroscopic Peritoneal Cancer Index (PCI) [6]. To gain more insight into the effect of PIPAC on the tumor cell composition of peritoneal metastases, we performed single cell karyotype sequencing (scKaryo-seq [8]) on biopsies obtained prior to each treatment cycle. After quality checks and omission of non-tumor cells with 2 N genomes (see methods section), this yielded 13 evaluable sequenced metastasis samples from cycle 1 (7 patients) 5 samples from cycle 2 (3 patients), 8 samples from cycle 3 (3 patients), and 2 samples from cycle 4 (1 patient) (Figure S1). In 11 cases samples were obtained from >1 timepoint from the same region, allowing analysis of the longitudinal effect of PIPAC on tumor cells obtained from the same regions. Qualitative analysis of the single cell karyotypes showed that in multiple metastases (03-M2; 04-M1; 04-M2; 05-M2; 17-M2;17-M3), subclones that were present in cycle 1, disappeared in cycle 2 or 3 (Figure S1). In 2 metastases from 1 patient, subclones (re-) appeared in later cycles (04-M1; 04-M3) (Figure S1).

**Fig. 1:**
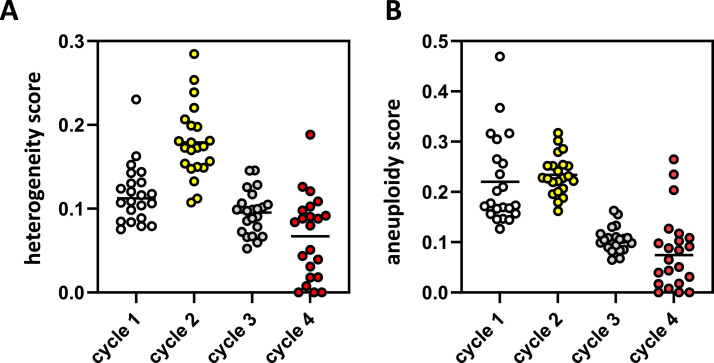
PIPAC reduces intra-tumor genetic heterogeneity and selects for cells with simpler karyotypes. Single cell shallow sequencing data (Figure S1) were analyzed for genetic heterogeneity scores (A) and aneuploidy scores (B). To visualize the longitudinal effect of PIPAC all scores of all available samples (Figure S2) were plotted over time. Cycle 1 contains 13 samples (regions/metastases) from 7 patients. Cycle 2 contains 5 samples from 3 patients. Cycle 3 contains 8 samples from 3 patients. Cycle 4 contains 2 samples from 1 patient. Each dot represents a single chromosome and displays the average heterogeneity and aneuploidy scores per chromosome (22 dots).

Single cell karyotype sequencing can also be used to calculate genomic heterogeneity, a measure for genomic diversity among individual cells from a single sample [8]. Keeping in mind the potential caveats of relatively low cell numbers and mixed populations in the biopsies, our analysis of the PIPAC sequencing results indicated that genomic heterogeneity among surviving tumor cells significantly increased after the first treatment cycle, but drastically dropped after the second and third treatment cycle (Fig. 1A). In addition, aneuploidy scores (a measure for karyotype complexity) also significantly dropped after the second and third treatment (Fig. 1B). Together, the data show that repetitive PIPAC likely changes the tumor cell composition of peritoneal metastases, yielding metastases with reduced intra-tumor heterogeneity and less complex karyotypes.

### PIPAC reduces hypoxia-related gene expression in peritoneal metastases

**Fig. 2:**
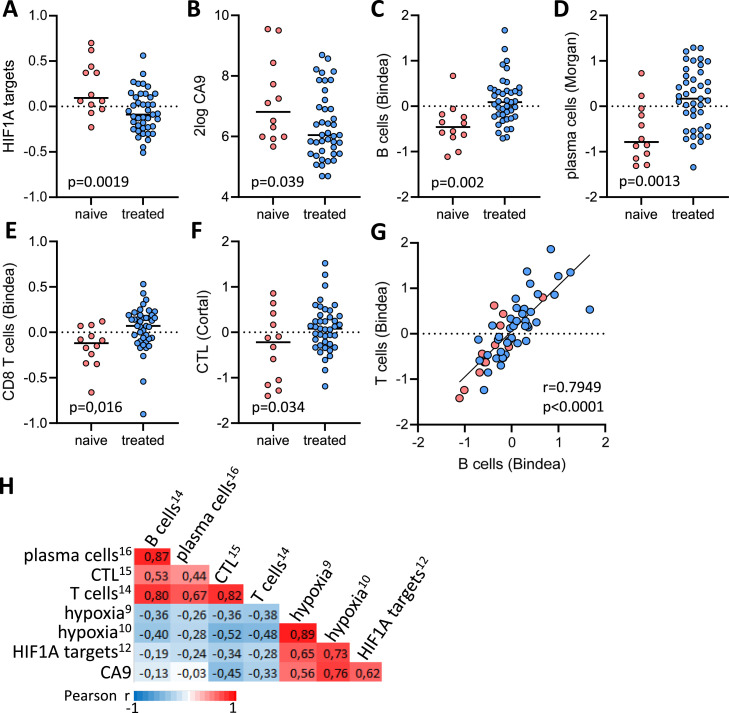
PIPAC reduces hypoxia-related gene expression and alters the immune microenvironment of peritoneal metastases. Dotplots showing expression of (A) HIF-1 alpha target genes [12] (B) CA9, (C) B cell signature [14], (D) plasma cell signature [16], (E) CD8 T cells [14], (F) CTLs [15] in treatment-naïve (red dots) versus PIPAC-treated (blue dots) peritoneal metastases. (G) XY plot showing the correlation between expression signatures reflecting B cell and T cell infiltration into peritoneal metastases. Treatment status is color coded: ePIPAC-naïve (red dots) and PIPAC-treated (blue dots. H) Heatmap showing the inverse relationship between expression of immune cell signatures and hypoxia-related gene expression. Pearson r values are color-coded from −1 (blue) to +1 (red). (I) CLUE-GO analysis of the 46 PIPAC-induced proteins identified by proteomics in both patient 3 and patient 6. See Table S4 for the complete protein list and Table S5 for statistical significance of the overrepresentation of GO terms and associated proteins in PIPAC-treated metastases.

From 53 samples we obtained high-quality RNA for subsequent analysis by RNA sequencing. Of these, 12 samples were from PIPAC treatment-naïve metastases, and 41 were from PIPAC-treated metastases. Expression of KEGG and Gene Ontology gene signatures reflecting tumor cell proliferation or apoptosis were not significantly different between PIPAC-naïve and treated samples (Figure S2A-D). In addition, immunohistochemistry on tumor biopsies using the proliferation marker Ki67 showed that all analyzed lesions were positive, and thus proliferative, regardless of treatment cycle (Figure S2E,F). Other cancer hallmarks, reflected by expression of 50 Cancer Hallmark signatures from the Molecular Signatures Database (MSigDB) [9], were also not significantly different between PIPAC-naïve and -treated groups (Table S2). However, expression of the Hallmark gene signature reflecting Hypoxia showed a near-significant (*p* = 0.065) decrease in expression following PIPAC (Table S2). Moreover, three additional signatures reflecting Hypoxia [[10], [11], [12]], Hypoxia-Inducible Factor 1-alpha (HIF1A) target genes [13], and the generic hypoxia marker CA9 were all significantly reduced following PIPAC. (Fig. 2A-B, Figure S3A-B, Table S2).

### PIPAC alters the immune microenvironment in peritoneal metastases

Hypoxia alters multiple aspects of the tumor microenvironment, and is a major driver of immunosuppression and resistance to immune checkpoint blockade [14]. Therefore, we analyzed expression of signatures reflecting the presence of various types of immune cells [15–17]. This revealed a marked upregulation of gene signatures derived from B cells, plasma cells, CD8 (cytotoxic) T cells (CTL), and activated dendritic cells in PIPAC-treated metastases (Fig. 2C-F, Table S3). Comparison of longitudinal samples revealed that expression of these signatures was induced after the first PIPAC cycle, and remained high afterwards (Figure S3C-F), while hypoxia signatures were inversely regulated over time (Figure S3A-B). Expression of signatures reflecting the presence of B cells, T cells, CTLs and plasma cells were strongly positively correlated (Fig. 2G-H). Of the 22 peritoneal metastasis samples with the highest expression of both T and B cell signatures (right upper quadrant in Fig. 2G), 21 were from PIPAC-treated metastases (95 %), while only one was from a treatment-naïve metastasis (5 %). Expression of PIPAC-induced immune cell signatures was strongly negatively correlated with expression of various hypoxia signatures and the hypoxia marker CA9 (Fig. 2H).

**Fig. 3:**
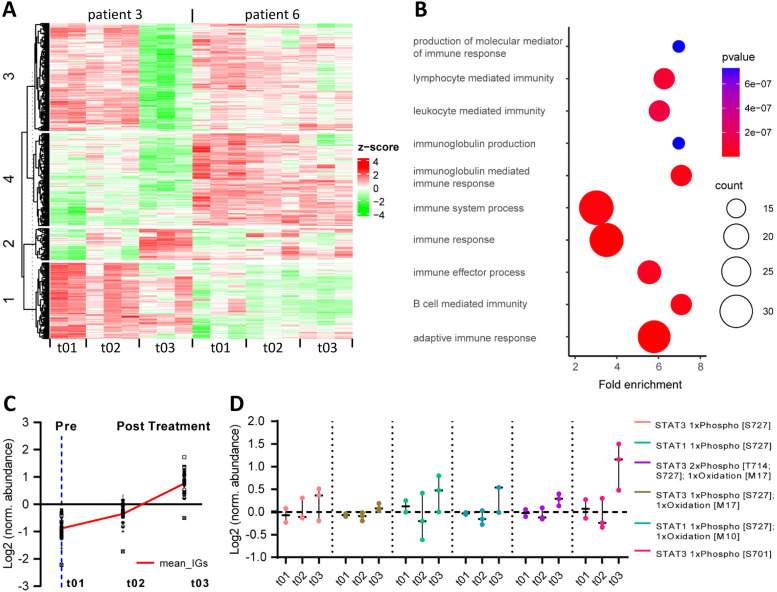
Proteomic and phosphoproteomic analysis of PIPAC-treated peritoneal metastases. (A) Unsupervised hierarchical clustering of 739 ANOVA-significant proteins based on z-scored log2 abundance values for patient 03 and 06 over the time-course of the treatment (t1, pretreatment, t2 and t3, both posttreatment). Patient 03 timepoint 1 (t1) is represented with *n* = 2. (B) Statistical overrepresentation test for proteins assigned to cluster 2 (patient 3) using GO-terms (biological process). Overrepresented terms are filtered for a Benjamini-Hochberg FDR < 0.05. (C) Time course of immunoglobuline protein expression. Log2-normalized protein abundances for 32 immunoglobulines grouped in cluster2 are shown. A mean immunoglobuline expression value for each timepoint was calculated and the trendline is indicated in red. (D) Selected time courses of STAT1 and STAT3 phosphopeptide isoform expression. Individual log2-normalized phosphopeptide isoform abundances per timepoint are shown, median values are indicated with horizontal dash (-). Patient 03 timepoint 1 (t1) is represented with *n* = 2.

Next, we analyzed peritoneal metastasis biopsies from two patients by proteomics. In total, 739 proteins showed a significantly different expression across the treatment course (Table S4). Unsupervised hierarchical clustering of these proteins generated 4 clusters (Fig. 3A). The abundance of proteins in one of these clusters (cluster 2) increased following PIPAC treatment (Fig. 3A). Analysis of Gene ontology (GO) terms revealed a statistical overrepresentation of proteins involved in B-cell mediated immunity and production of immunoglobulins in this treatment-induced cluster 2 (Fig. 3B, Table S5). A major fraction of the proteins in cluster 2 (32/76) consists of immunoglobulin heavy chains (*n* = 17) and immunoglobulin light chains (lamba, *n* = 7; kappa, *n* = 8) (Fig. 3C, Table S6). This is in accordance with the RNA sequencing data, showing a significantly higher expression of signatures reflecting the presence of B cells and plasma cells following PIPAC treatment (Fig. 2C-D).

The above results suggest that PIPAC may have an immune-priming effect on peritoneal metastases. Signal Transducer and Activator of Transcription proteins (STATs) are central regulators Aof immune cell function, and are activated by phosphorylation [18]. Phospho-proteomics analysis of the biopsies taken prior to and following PIPAC revealed that phosphorylation of serine 727 in STAT1 and STAT3, a STAT-activating phosphorylation site [19], was increased following PIPAC (Fig. 3D, Table S7). In addition, PIPAC induced phosphorylation of Ser701 in STAT3 (Fig. 3D, Table S7), which causes STAT dimerization and activation [20].

### PIPAC reduces expression of immunosuppressive cytokines and increases expression of immune checkpoints

**Fig. 4:**
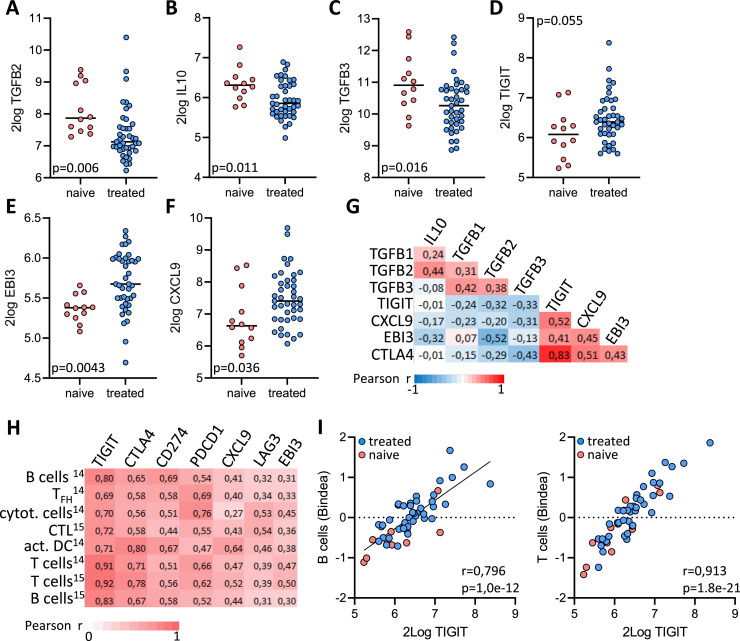
PIPAC causes reduced expression of immunosuppressive cytokines and increased expression of immune checkpoint genes. Dotplots showing RNA expression levels of (A) TGFB2, (B) IL10, (C) TGFB3, (D) TIGIT, (E) EBI3, and (F) CXCL9, in treatment naïve peritoneal metastases versus all PIPAC-treated samples. See Table S7 for the complete gene list examined. (G) Heatmap showing the inverse relationship between expression of immunosuppressive cytokines (IL10, TGFB2, TGFB3) and immune checkpoint genes (TIGIT, CXCL9, EBI3). Pearson r values are color-coded from −1 (blue) to +1 (red). (H) Heatmap showing the positive correlation of expression of signatures reflecting immune cell infiltration and checkpoint activation. Pearson r values are color-coded from 0 (white; no correlation) to +1 (red; perfect correlation). (I) XY plots showing the correlation between expression of signatures reflecting B cell (left) and T cell (right) infiltration with expression of the immune checkpoint gene TIGIT.

Immunosuppression in the tumor microenvironment is mediated by cytokines that prevent the generation of cytotoxic T lymphocytes (CTL), and by activation of immune checkpoints that protect tumor cells from CTLs, in case they are generated [21]. To gain insight into how PIPAC may alter these signals we compiled a list of 39 immunosuppressive cytokines, enzymes, checkpoints and their ligands [21] (Table S8), and assessed how PIPAC altered their expression. This analysis revealed that PIPAC significantly reduced the expression of immunosuppressive cytokines (TGFβ ligands, interleukin-10) (Fig. 4A-C; Table S8) and simultaneously increased expression of the checkpoints EBI3 (the interleukin-27 beta subunit), TIGIT and the IFNγ-induced T cell-recruiting chemokine CXCL9 (Table S8, Fig. 4D-F). Interestingly, CXCL9 is present in 9 distinct signatures predicting response to immune checkpoint inhibition [22]. In general, there was an inverse correlation between expression of immunosuppressive cytokines (high in PIPAC-naïve samples) and genes encoding immune checkpoints (high in PIPAC-treated samples) (Fig. 4G). There was also a highly significant correlation between expression of various B- and T-cell signatures and expression of the immune checkpoint gene TIGIT (*r* = 0.80, *p* = 1,0e-12 and *r* = 0.91, *p* = 1.8e-21 respectively) (Fig. 4H). The vast majority of samples expressing high levels of both TIGIT and B- or T-cell signatures were derived from PIPAC-treated metastases (9/10 and 10/12 respectively). Highly significant and strong positive correlations were observed between expression of signatures reflecting influx of B cells, (cytotoxic) T cells, T follicular helper cells, activated dendritic cells, and various immune checkpoint genes (Fig. 4H-I). Together, the data indicate that PIPAC reduces immunosuppressive signals in peritoneal metastases (hypoxia, IL10, TGFB), and causes an influx of B and T cells, and subsequent activation of immune checkpoints.

### PIPAC induces the formation of metastasis-associated tertiary lymphoid structures (TLS)

**Fig. 5:**
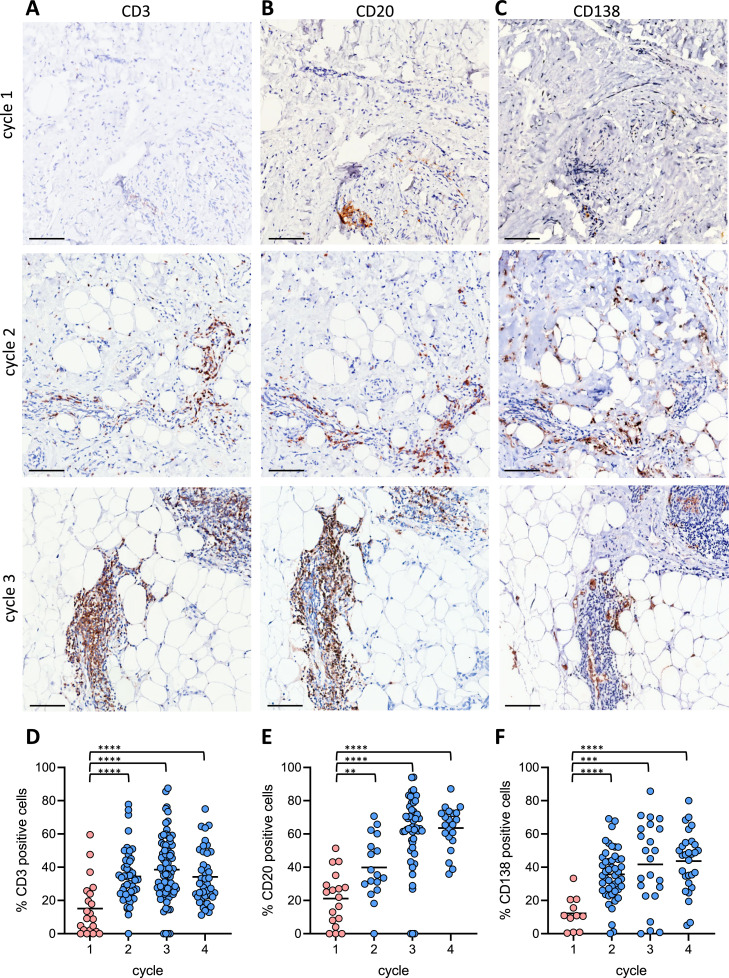

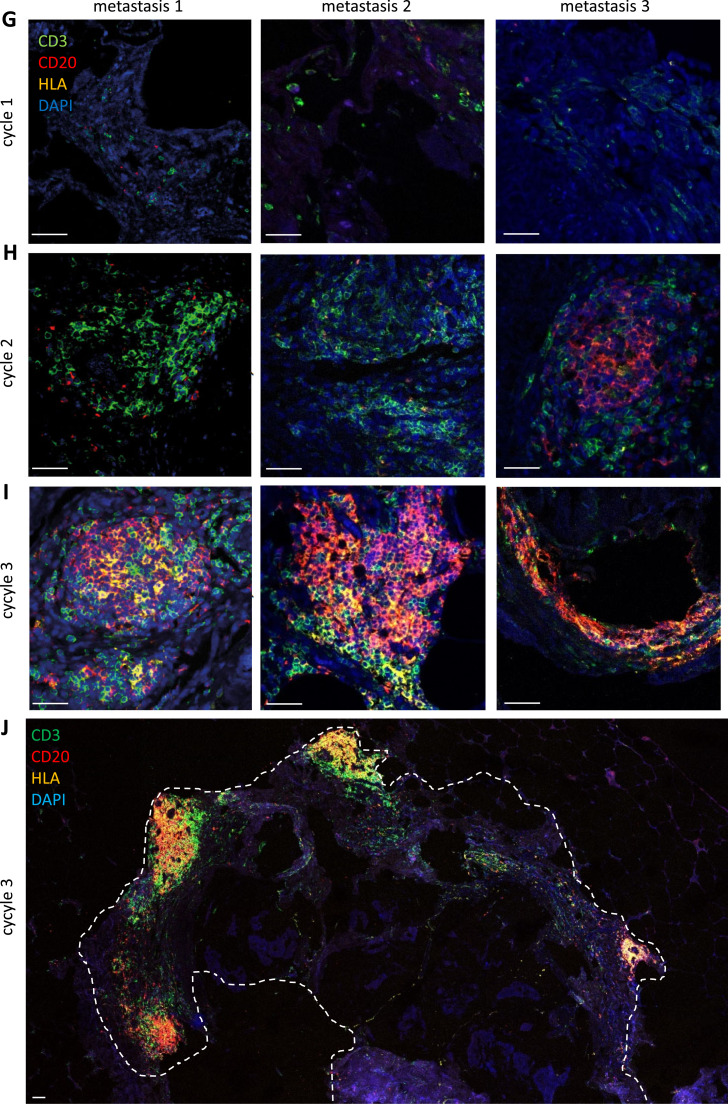
PIPAC causes formation of metastasis-associated Tertiary Lymphoid Structures (TLS). Immunohistochemistry analysis of (A) CD3 (T cells), (B) CD20 (B cells), (C) CD138 (plasma cells) in biopsies from PIPAC-naïve and -treated samples over time. All stained sections were quantified using QuPath, and the resulting data are represented in (D) CD3, (E) CD20, and (F) CD138. (G) Immunofluoresence analysis of the expression of CD3 (T cells), CD20 (B cells) and HLA (MHC class II) in three peritoneal metastases over time. Low expression of all three markers in PIPAC-naïve metastases (cycle 1). Clusters of B and T cells in ePIPAC-treated metastases start to form after one (B) and two (C) treatment cycles. (J) Overview of a metastasis (dotted white line) surrounded by various TLS following 2 cycles of PIPAC. Bar=250μm. Significance of the differential staining between treatment cycles was assessed using student’s T test. **p < 0.001. ***p < 0.0001. ****p < 0.00001. Bar=250 mm.

To validate the above findings, we performed immunohistochemistry (IHC) using anti CD3 (T cells), anti CD20 (B cells) and anti CD138 (plasma cells) antibodies. In PIPAC-naïve samples, the number of metastasis-infiltrated T cells, B cells and plasma cells was very low (Fig. 5A-C). However, this number significantly increased following PIPAC (Fig. 5A-C; quantified in Fig. 5D-F). Moreover, while immune cells were scattered in PIPAC-naïve samples they clustered together into immune cell aggregates following PIPAC-exposure (Fig. 5A-C). A specific form of immune cell aggregates are tertiary lymphoid structures (TLS). TLS formation in and around tumors has recently gained attention for their presumed role in the generation of anti-tumor immune responses [23]. TLS are characterized by the presence of High Endothelial Venules (HEV), acting as entry sites for immune cells [24]. Immunohistochemistry for the HEV marker MECA-79 revealed that the immune cell aggregates found in PIPAC treated peritoneal metastases indeed contained HEVs (Figure S4). TLS are also characterized by juxtaposed zones of B and T cells. To analyze the relative position of B cells and T cells in PIPAC-induced immune cell aggregates we performed multiplexed immunohistochemistry using anti-CD20 (B cells) and anti-CD3 (T cells) and fluorescent secondary antibodies. In treatment-naïve tissues, co-localization of the low numbers of scattered B and T cells was very limited (Fig. 5G). Following PIPAC, B-cells and T-cells co-clustered into metastasis-surrounding TLS with juxtaposed regions of both cell types (Fig. 5G-J). One of the presumed roles of B cells within tumor-associated TLS is the presentation of tumor antigens to nearby T cells. Indeed, the majority of B cells in PIPAC-induced TLS expressed Major Histocompatibility Class II (encoded by the Human Leukocyte Antigen (HLA)) on their cell surface and were in close proximity to T cells (Fig. 5G-J).

### PIPAC triggers plasma cell formation and the generation of tumor-reactive antibodies

B cells within TLS may act as antigen-presenting cells (Fig. 5), but they may also differentiate into antibody-producing plasma cells. Indeed, the RNAseq data (Fig. 2), the proteomics data (Fig. 3, Table S6) and the IHC data (Fig. 5) indicate that PIPAC increases the number of plasma cells within peritoneal metastases. Analysis of ascites samples for the presence of specific Ig subtypes by ELISA showed that PIPAC caused a marked increase in the levels of IgG1, IgG3 and IgG4 in patients 12, 13 and 17 (Figure S5A). IgG 2 also increased following PIPAC in patients 13 and 17 (Figure S5A). No such PIPAC-induced increase was observed for IgA1, IgA2 or IgM. Patient 12 showed the strongest increase in IgG content following PIPAC. The samples from this patient were chosen to study potential PIPAC-induced changes in the repertoire of antigens recognized by ascites-derived antibodies. To this end the ascites samples from PIPAC-naïve and -treated patient 12 were analyzed for reactivity against a human protein array containing 23,133 purified protein spots representing the human proteome. This analysis revealed that PIPAC-exposure strongly increased the number of antigens recognized by ascites-derived antibodies (Figure S5B; Table S9). The targets identified include secreted proteins, proteins expressed on the cell surface as well as intracellular proteins (Table S9). To test whether ascites-derived antibodies can recognize tumor cells, we stained tumor organoids from patient 3 with ascites collected before and after PIPAC from the same patient, using immunofluorescence and confocal microscopy. Tumor-reactivity was observed when using ascites collected after the first PIPAC procedure just prior to cycle 2, but not when using ascites that was collected before PIPAC (prior to cycle 1) (Figure S5C).

## Discussion

An extensive biopsy protocol followed by genetic and molecular analysis of the resulting tissue biobank revealed two major effects of PIPAC on peritoneal metastases. First, repetitive treatment caused a reduction of intra-tumor genetic heterogeneity. Moreover, treatment-surviving tumor cells had genomes with reduced karyotype complexity. Interestingly, we recently demonstrated that resistance to radiation in rectal cancer organoids is associated with reduced chromosomal instability [25]. Possibly, tumor cells with complex karyotypes and chromosomal instability may be especially sensitive to irradiation and to DNA-damaging agents such as oxaliplatin. These genetic results are in line with the observed anti-tumor effect of the PIPAC procedure, evidenced by reduced PCI and PRGS scores [26]. However, the PIPAC-surviving tumor cells remain proliferative, causing continued growth of PM despite clone selection. Future studies in larger patient cohorts are required to assess how intra-PM genetic heterogeneity and/or clone selection during treatment are correlated with survival. Higher levels of heterogeneity would increase the chance that treatment-resistant subclones already exist prior to treatment.

Second, PIPAC caused an influx of T cells, B cells and plasma cells, and their organization into tertiary lymphoid structures (TLS). Tumor-associated TLS act as platforms for the generation of an anti-tumor immune response, mediated by B and T cells [23]. The induction of TLS following chemotherapy has also been observed in primary breast cancer [27], pancreatic adenocarcinoma [28], hepatoblastoma [29], and peritoneal mesothelioma [30]. TLS induction likely depends on the ability of specific drugs to induce immunogenic cell death (ICD) [31]. Interestingly, oxaliplatin is one of the few chemotherapeutic drugs causing ICD, in which dying tumor cells present tumor-specific antigens and elicit an adaptive anti-tumor immune response [31].

B cells residing in TLS may play multiple distinct roles in shaping the tumor immune microenvironment. Mature TLS support B cell maturation and differentiation into IgG- and/or IgA-producing plasma cells [23]. Analysis of ascites samples revealed that PIPAC caused an increase in the levels of IgG1–4 and IgA1, but not IgM, in multiple patients, reflecting the process of immunoglobulin class switching and plasma cell generation. Analysis of ascites samples and tumor organoids showed that the antibodies generated after PIPAC could recognize tumor-associated antigens (TAA). Interestingly, previous research showed that plasma cells and TAA-reactive antibodies can also be detected in ovarian tumors and peritoneal metastases, where they correlate with better prognosis [[32], [33], [34]]. TAA-IgG complexes may be taken up by dendritic cells and contribute to antigen presentation and T cell-mediated tumor cell killing [35]. Alternatively, antibodies bound to TAAs on the tumor cell surface may result in direct tumor cell killing by natural killer (NK) cells in antibody-dependent cellular cytotoxicity (ADCC) [36]. Finally, tumor-bound antibodies may result in local activation of the complement system, which could lead to complement-dependent cytotoxicity (CDC) or, in case the tumor cells are CDC-resistant, to chronic local inflammation and tumor progression [23]. Upon antigen recognition by the B cell receptor, B cells may also internalize the TAA and process it into small peptides for subsequent presentation to neighboring T cells in the context of MHC class II. Indeed, we found that the B cells within PIPAC-induced TLS express MHC class II and are in close proximity to T cells. Taken together, B cells are likely to play major and diverse roles in PIPAC-induced changes in the PM immune microenvironment. Future work should elucidate which of these processes are most important in determining oncological outcome in these patients, and which of them may be exploited for the design of effective combination treatment strategies.

In general, the presence of B cells and tumor-associated TLS correlates with a better prognosis in multiple cancer types including CRC [24,[37], [38], [39]], and predicts responsiveness to immune checkpoint inhibitors such as anti-PD1 or anti-CTLA4 [[40], [41], [42], [43]]. Response to immune checkpoint inhibitors in metastatic colorectal cancer (mCRC) is generally only observed in tumors with a high tumor mutational burden (TMB) caused by a deficient mismatch repair system (dMMR) [44,45]. This CRC subtype is enriched in patients with peritoneal metastases [46]. However, none of the patients included in the current PIPAC study had dMMR tumors. Interestingly, neoadjuvant treatment of primary CRC shows that tumors with a proficient MMR system (pMMR) can also respond to immune checkpoint inhibitors [47]. Therefore, the lower mutational load in pMMR versus dMMR CRC does not preclude their response to immune checkpoint inhibitors *per se.* Indeed, a recent phase three study in metastatic gastric and esophageal carcinoma, cancer types with a similar TMB compared to CRC [48], demonstrated that the combination of anti-PD1 with oxaliplatin-containing chemotherapy regimens was superior over chemotherapy alone [49]. The patients in this study were not pre-selected according to tumor MMR status. Recently, the results of the PIANO trial were published, a Phase-1 study evaluating PIPAC with oxaliplatin and anti-PD1 (nivolumab) in gastric cancer patients with inoperable PM [50]. This study demonstrated that the combination treatment was safe and that it induced an influx of naïve CD8 T cells into the PM and a reduction of M2 macrophages and NK T cells [50]. Combination treatment caused a reduction of the PCI and demonstrated pathological responses (i.e. reduced PRGS scores), but this did not lead to improved survival [50]. In our PIPAC study [26], response (anti-tumor efficacy) was measured in several ways: radiological, biochemical, pathological (PRGS), cytological, and macroscopic. Interestingly, as in the PIANO study, we observed a reduction of the PCI and PRGS following PIPAC, but this did not correlate with other response measures and did not translate into survival benefit. Nevertheless, the current study and the PIANO trial both show that PIPAC-oxaliplatin has a stimulating effect on the adaptive immune system in PM and that the combination of PIPAC-oxaliplatin with immune checkpoint blockade deserves further clinical evaluation.

The presence of peritoneal metastases and the formation of ascites is associated with a poor response to immune checkpoint inhibition, both in colorectal cancer patients and in mouse models [[51], [52], [53]]. The therapeutic induction of TLS formation is now considered an attractive strategy to sensitize tumors to immune checkpoint inhibitors [23]. The results presented in this report demonstrate that, in the context of colorectal peritoneal metastases with ascites, 1–2 cycles of intraperitoneal delivery of oxaliplatin through PIPAC, is sufficient to induce the formation of metastasis-associated TLS.

The major strength of our study is the temporal nature of the multi-level analysis of PM in cancer patients under treatment. Longitudinal analysis of distant metastases under treatment is generally very difficult, due to the inaccessibility of metastatic tissue. The cyclic nature of intra-abdominal treatment with PIPAC uniquely allows ‘real-time’ monitoring of how treatment alters the biology of PM and their (immune) microenvironment. A limitation of our study is the descriptive nature of the results. As such, we cannot be sure whether the observed changes in the immune TME are indeed sufficient to prime PM to respond to immune checkpointing inhibitors. Indeed, the combination of PIPAC with pembrolizumab did not improve survival in gastric cancer patients with PM [50]. Therefore, follow-up studies will include finding the most potent ICD-inducing drug and the most relevant immune checkpoint to target, by applying pre-clinical models based on PM-derived organoids [54] and their use in humanized mice [51].

In conclusion, our results show that PIPAC causes major changes in the immune microenvironment of PM and induces biomarkers that predict responsiveness to immune checkpoint inhibition. Therefore, we propose that immune checkpoint inhibition in patients receiving PIPAC treatment for peritoneal metastases may yield (potentially lasting) clinical benefit. Given the repeated access to the peritoneal cavity that is required for the PIPAC procedure, this would provide a unique opportunity to test whether this novel treatment strategy indeed leads to the generation of local and systemic anti-tumor immunity, and to identify the specific immunological processes that would constitute such a response.

## Data availability

Sequencing and proteomics data are available through the relevant repositories. Other data will be made available upon reasonable request by the corresponding authors.

## Funding

This study was funded through a kind donation of the Dalijn foundation.

## CRediT authorship contribution statement

**Alexander Constantinides:** Writing – review & editing, Writing – original draft, Project administration, Investigation, Formal analysis. **Nico Lansu:** Writing – original draft, Methodology, Formal analysis. **Peter Mosen:** Writing – original draft, Formal analysis. **Paulien Rauwerdink:** Formal analysis. **Esther Strating:** Formal analysis. **Franziska Völlmy:** Formal analysis. **Maaike Nederend:** Formal analysis. **Jeanette H.W. Leusen:** Methodology. **Koen Rovers:** Resources. **Emma Wassenaar:** Resources. **Robin Lurvink:** Resources. **Maarten Altelaar:** Supervision, Methodology. **Simon Nienhuijs:** Resources. **Rene Wiezer:** Resources. **Inne H.M. Borel Rinkes:** Supervision, Funding acquisition. **Djamila Boerma:** Supervision, Resources. **Geert J.P.L. Kops:** Supervision, Methodology. **Ignace de Hingh:** Supervision, Resources, Conceptualization. **Onno Kranenburg:** Writing – review & editing, Writing – original draft, Supervision, Funding acquisition, Conceptualization.

## Declaration of competing interest

The authors declare that they have no known competing financial interests or personal relationships that could have appeared to influence the work reported in this paper.

## Appendix Supplementary materials

Image, application 1
