# Supplementary material for: Treatment of colorectal peritoneal metastases with oxaliplatin induces biomarkers predicting response to immune checkpoint blockade
Source: Transl Oncol. 2025 Jul 1;59:102464. doi: 10.1016/j.tranon.2025.102464 (PMC12269567; doi:10.1016/j.tranon.2025.102464)
Supplement: Supplementary file 1 [file mmc1.docx]

**Treatment of colorectal peritoneal metastases with oxaliplatin induces biomarkers predicting response to immune checkpoint blockade**

Alexander Constantinides^1,2^, Nico Lansu^3,4^, Peter Mosen^5^, Paulien Rauwerdink^1,2,6^, Esther Strating^1,2^, Franziska Völlmy^5^, Maaike Nederend^7^, Jeanette H. W. Leusen^7^, Koen Rovers^8^, Emma Wassenaar^1,2,7^, Robin Lurvink^8^, Maarten Altelaar^5^, Simon Nienhuijs^8^, Rene Wiezer^6^, Inne H.M. Borel Rinkes^1,2^, Djamila Boerma^6^, Geert J.P.L. Kops^3,4^, Ignace de Hingh^8,9*^, Onno Kranenburg^1,2,10*^

^1^Laboratory Translational Oncolo­­­gy, Division Imaging and Cancer, University Medical Center Utrecht; Utrecht, The Netherlands.

^2^Department of Surgical Oncology, Division Imaging and Cancer, University Medical Center Utrecht; Utrecht, The Netherlands.

^3^Hubrecht Institute, Royal Netherlands Academy of Arts and Sciences (KNAW), and University Medical Center Utrecht, Utrecht, The Netherlands.

^4^Oncode Institute, Utrecht, The Netherlands,

^5^Biomolecular Mass Spectrometry and Proteomics, Bijvoet Center for Biomolecular Research, Utrecht Institute for Pharmaceutical Sciences, Utrecht University and Netherlands Proteomics Centre, The Netherlands

^6^Department of Surgery, Antonius Hospital; Nieuwegein, The Netherlands

^7^Center for Translational Immunology, University Medical Center Utrecht; Utrecht, The Netherlands

^8^Department of Surgery, Catharina Hospital; Eindhoven, The Netherlands

^9^Department of Epidemiology, GROW-School for Oncology and Developmental Biology, Maastricht University, Maastricht, The Netherlands

^10^Utrecht Platform for Organoid Technology, Utrecht University; Utrecht, The Netherlands

***Corresponding authors:** Ignace de Hingh, Department of Surgery, Catharina Hospital; Eindhoven, The Netherlands. Phone: +31-40-2396600. Fax: Email: [ignace.d.hingh@catharinaziekenhuis.nl](mailto:ignace.d.hingh@catharinaziekenhuis.nl)

Onno Kranenburg, Laboratory of Translational Oncology, Division Imaging and Cancer, University Medical Center Utrecht; 3584CX, Utrecht, The Netherlands. Phone: +31-88-7558632. Fax: +31-88-7569910. Email: [o.kranenburg@umcutrecht.nl](mailto:o.kranenburg@umcutrecht.nl). ORCID ID: 0000-0002-2112-4390

| **Supplementary Methods** |  |
| --- | --- |
| **Manuscript preparation**  AI tools were not used during the preparation of this manuscript. Relevant literature was identified in PUBMED using search terms like ‘colorectal’, ‘pipac’, ‘oxaliplatin’, peritoneal metastasis’, ‘tertiary lymphoid structure’, etc. in various combinations.  **Single cell karyotype sequencing**  Frozen Pipac biopsy samples were processed for sorting by cutting a small piece of snap-frozen tissue which was mixed with 1 ml of Nuclei Suspension Buffer (100 mM Tris-HCl pH 7.5, 154 mM NaCl, 1 mM CaCl2, 0.5 mM MgCl2, 0.2% BSA, 0.1% NP40 Alternative (Santa cruz), 1 ug/mL Hoechst 34580 (Sigma-Aldrich)) inside a petri dish, and minced on ice, using a cross-hatching motion with two scalpels. The minced tissue was kept on ice for 30 min after which it was filtered through a 70-μm strainer into a FACS tube. Single G1 Nuclei were sorted on a BD FACSAria Fusion Flow Cytometer from the Hubrecht Flow Cytometry Core facility. In a 384-well plate containing 5μl mineral oil (Sigma-Aldrich) in each well and stored at −20 °C until further processing.  NlaIII-based library preparation: Mixes were dispersed with the Nanodrop II liquid handling platform (GC biotech). Cell lysis was performed in 100 nl of 1× CutSmart buffer (NewEngland Biolabs) containing 2 mg/ml Proteinase K (Fisher Scientific) at 55°C for 2 h and heat inactivated at 80°C for 15 min. The genomic DNA was subsequently fragmented in 100 nl of 1× Cutsmart buffer (New England Biolabs) containing 1 U/µl NlaIII (New England Biolabs) at 37°C for 2 h and heat inactivated at 80°C for 20 min. 50 nl of 100 nM barcoded, double-stranded NLAIII adaptors (adaptors consisted of a NLAIII sequence, a 3bp random molecular barcode (UMI), a cell-specific 8bp barcode, the 5′ Illumina TruSeq small RNA kit adaptor and a T7 promoter) were ligated with 150 nl of 1× T4 DNA ligase buffer (New England Biolabs) containing 66 U/µl T4 DNA ligase (New England Biolabs), supplemented with 3 mM ATP (Invitrogen) at 16°C overnight. Aqueous phase was separated from the oil phase after pooling all cells in one reservoir, DNA purification was preformed using AMPure XP magnetic beads (Agencourt AMPure, Beckman Coulter, Brea, CA, USA) followed by IVT transcription with the MegaScript T7 Transcription Kit (ThermoFisher). Amplified RNA of each pool was then reverse transcribed, using randomhexRT primers (GCCTTGGCACCCGAGAATTCCANNNNNN) which contain the illumina RNA RT primer sequence using Superscript II (ThermoFisher). Illumina sequencing libraries were then prepared with the TruSeq small RNA primers (Illumina) using Thermo Scientific PCR Master Mix followed by a final DNA purification using AMPure beads.  Samples were sequenced on the llumina NovaSeq 6000 and NextSeq2000 platforms at 1× 100 base pairs (bp) length. After sequencing, fastq files were mapped to GRCH38 using the Burrows–Wheeler aligner (bwa aln 0.7.12 and python 2.7.5) and parsed for library barcodes, removed reads without an NlaIII sequence and removed PCR-duplicated reads using the UMI. Analysis of copy number variation (CNV) patterns of single cells were performed using the R package Aneufinder (v.1.14.0). Edivisive copy number calling was used with bins of 5 Mb, and a custom blacklist strategy to exclude reads from artefact-prone regions. After single-cell copy number calling with Aneufinder, we excluded cells with low quality sequencing data (spikiness <0.21 and Bhattacharyya distance >1.0) from further analysis through use of a custom R script named AneufinderFileFilter (https://github.com/TWvR/AneufinderFileFilter). This script was also used to remove perfect diploid cells from our analyses, since these most likely represent non-cancerous cells. (All cells that had the following three characteristics were removed; Segment length =1, segment copy number state=2, x chromosome copy number state =1 (male) or =2 (female)). The script also created all genomewide heterogeneity and aneuploidy plots of the chromosome measures used in the analysis.  **RNA sequencing**  Biopsies were thawed and lysed using TRIzol Reagent (Invitrogen, 15596026) and the Precellys 24 Tissue Homogenizer (Hyland Scientific) and tissue homogenizing beads (Bertin instruments, P000918-LYSK0-A), followed by theTRIzol Reagent manufacturer’s protocol. RNA samples were stored at -80 ^0^C in RNase-free water. RNA quality control was performed with the Bioanalyzer (Agilent, G2939BA). RNA quantity was analyzed using the Qubit RNA Broad Range Assay Kit assay (Invitrogen, Q10210) on the Qubit Flex Fluorometer (Invitrogen, Q33327) according to the manufacturer’s protocol. Library preparation and Next Generation Sequencing was performed by USEQ (Utrecht Sequencing Facility, Utrecht, The Netherlands) with standard Illumina (Illumina, San Diego, CA USA) protocols. For the generation of sequencing libraries after ribosomal RNA depletion with the Illumina Ribo-Zero Plus rRNA Depletion Kit (Illumina), Truseq RNA stranded polyA (Illumina) was used. Samples were sequenced on the platform Illumina NextSeq2000 with 2 x 50 bp (Illumina).    **Immunohistochemistry on biopsies**  Paraffin embedded tissue from CRC-PIPAC patients was cut into sections which were deparaffinized and hydrated in xylene and ethanol series. Endogenous peroxidase activity was blocked with 10 minutes of hydrogen peroxide block. Slides were cooked in antigen retrieval buffer for 20 minutes, afterwards buffer and slides were left to cool off for 20 minutes. Antibodies were diluted in PBS Azide BSA buffer and added to the slides. Slides were left to incubate overnight at 4 deg C. After washing, secondary antibodies were added and were left to incubate for 1 hour at room temperature. After washing, the slides were incubated with DAB-H_2_O_2_ buffer for max 10 minutes. After washing, a hematoxylin staining was performed and slides were washed, dehydrated and covered mechanically. Antibodies used: CD3 (#ab5690 Abcam, citrate 1:200 overnight); CD20 (#48750 Cell signaling, citrate 1:400 overnight); CD138 (#AF2780 Citrate 1:50 overnight); MECA-79 (#NB100-77673 Novus Biologicals, EDTA 1:100 overnight), Tigit (#ab243903 Abcam citrate 1:150 overnight), EBI3 (#15k8D10 Novus Biologicals 1:100 citrate overnight); Secondaries: anti-rabitHRP (#K4003 1:1000 30 min); anti-ratHRP (#STAR72 1:50 30 min).  **LC-MS/MS sample preparation**  Biopsies from 3 patients undergoing PIPAC treatment were taken just prior to the first second and third PIPAC treatment, from the same metastasis locations. Samples were kept on ice in a detergent-based lysis buffer (1% sodium deoxycholate (SDC), 10 mM tris(2-carboxyethyl)phosphine (TCEP), 10 mM Tris, and 40 mM chloroacetamide) also containing EDTA-free protease inhibitor cocktail and phosphoprotease inhibitor tablets (Roche). A metal pestle was used to disrupt the tissue and the sample was heated at 95°C for 5 minutes. Protein extraction beads (cat. No C20000021, Diagenode) were added to the sample prior to sonication using a Bioruptor (30 cycles of 30sec on/30sec off, Diagenode). Samples were centrifuged at max speed for 10 minutes at 4°C and the supernatant was collected. 100µg of each sample was digested overnight at 37°C using trypsin (Promega) and LysC (both Wako) at 1:50 and 1:75 enzyme to substrate ratios, respectively. The digestion was quenched with 10% formic acid and the resulting peptides were cleaned-up in an automated fashion using the AssayMap Bravo platform (Agilent Technologies) with corresponding AssayMap C18 reverse-phase columns (C18 5μl, Agilent Technologies) followed by vacuum drying. In order to generate a reference channel to be used for all experiments, a pool composed of remaining samples (when feasible) was digested. Dried peptides were resolubilized in TMT resuspension buffer (87.5% HEPES, 12.5% ACN) and TMT10plex isobaric label reagents (cat.no 90406, Thermo Fisher) were prepared according to manufacturer’s instructions. TMT labels were added to samples and labeling was allowed to occur during 1.5 hours at RT after which the reaction was quenched using 5% hydroxylamine in HEPES, for 15 minutes at RT. The various channels were combined for each experiment and the acetonitrile content was reduced by evaporation. The samples were then cleaned (SepPak 1cc cartridge, 100mg, Waters) and dried completely before resolubilizing in HpH buffer A (10mM NH4OH, pH 10.8). HpH fractions were collected every minute over a 100 minutes gradient. Fractions between minutes 10 and 70 were concatenated into 20 fractions. An aliquot of each fraction was vaccum-dried and resuspended in 2% FA for proteomic data acquisition. Remaining fraction volumes were merged into 10 fractions, dried by SpeedVac and resuspended in IMAC enrichment buffer (80% ACN, 0.1% TFA). Enrichment of phosphopeptides was performed on an AssayMap Bravo platform (Agilent Technologies) following the standard protocol using Fe(III)-NTA Assay Map catridges (cat. No 5496-60085, Agilent Technologies). Recovered eluate was dried and enriched phosphopeptides were resuspended in 50mM citric acid for LC-MS/MS data acquisition.  **LC-MS/MS data acquisition**  Data of proteome samples were acquired on a Fusion Lumos Tribrid mass spectrometer, phospho proteome data were acquired on an Q Exactive HF-X Quadrupole-Orbitrap mass spectrometer system (both Thermo Fisher Scientific). Mass spectrometers were coupled on line to 1290 Infinity II LC systems (Agilent). Peptides resuspended in 0.1% FA (solvent A) or 50 mM citrate (phosphopeptide enriched samples) were trapped on a C18 PepMap100, 5 μm, 100 Å pre-column (300 μm i.d. x 5mm; Thermo Scientific) and then separated on a 50 cm (75 µm ID) in-house packed column using Poroshell 120 EC-C18 2.7-micron material (ZORBAX Chromatographic Packing; Agilent). For the analysis of phospho-enriched samples the LC system was equilibrated with multiple blank injections (50 mM citrate). Samples were eluted with linear gradients ranging from 12-40% (proteome samples) and 9-34% (phospho proteome samples) solvent B (80% ACN, 0.1% FA) over 125 minutes, 40% resp. 34%-100% solvent B for 3 minutes, followed by 100% solvent B for 1 minute at a flow rate of 200 µl/min. On the Fusion Lumos MS instrument MS1 scans were carried out at a resolution of 60, 000 in the orbitrap, covering 375-1500 m/z with an AGC target set to 4e5 and a maximum injection time of 50 ms. The TOP12 multiple charged peptides (charges 2+ to 6+) passing an intensity threshold of 5.0e4 counts were selected for subsequent isolation (quadrupole, 0.7 m/z isolation width, dynamic exclusion 20s), fragmented in HCD-mode (CE 38%) and dependent MS2 scans in the orbitrap followed. Fragment ion spectra were recorded at a resolution of 50,000 starting at 120 m/z, adjusting the AGC target to 5e4 and the maximum injection time to 90 ms. For the analysis of enriched phospho peptides using the Q Exactive HF-X instrument MS1 scans were performed in the orbitrap at a resolution of 60,000 covering 375-1500 m/z, setting the AGC target to 3e6 counts and a maximum injection time of 20 ms.TOP15 precursor ions with charges +2 to +5 were isolated (isolation width 1.2 m/z) and fragmented (HCD, 32% CE). Fragment ion spectra were recorded at a resolution of 45,000 covering 120–2,000 m/z. The AGC target was set to 1e5 (minimum AGC 1e4) and maximum injection time of 100 ms was defined. Dynamic exclusion was kept at 20 ms.  **Analysis and processing of LC-MS/MS data**  Data were analyzed with Proteome Discoverer (version 2.4.1.15; Thermo Fisher Scientific) using the Mascot search engine (version 2.8.0; Matrix Science). Database searches were performed against the SwissProt *Homo Sapiens* proteome (20,423 entries, release 2022/07) and the MaxQuant contaminant list. Search settings were as followed: trypsin as protease allowing up 2 missed cleavage sites, 20 ppm (MS1) resp. 0.05 Da (MS2) mass tolerance, Carbamidomethyl (C) and TMT10plex-tags (K and N-term) as static modifications, Oxidation (M) for proteome samples or Oxidation (M) and Phospho(STY) for phospho enriched samples as dynamic modification. Percolator was used for q-value-based validation of identified peptide spectrum matches (PSMs) applying a target FDR of 1%, resp. 5% (strict/relaxed). For localization of modification sites within validated peptide sequences IMP-ptmRS node^1^ was included to the workflow (PhosphoRS Mode: False, remaining settings as default). High confident PSMs were combined, aggregated to peptides and only high confident peptides (FDR < 1%) were used for protein grouping (principle of strict parsimony). TMT reporter ion extraction was performed with default settings (20 ppm integration tolerance; most confident peak integration). For reporter ion-based quantification following settings were applied: unique peptides exclusively, reporter ion abundance (readout) based on: S/N, apply Quan correction, co-isolation threshold: 50%, average reporter S/N threshold: 10, use all peptides for protein quantification/calculation. Further data processing of Proteome Discoverer outputs was performed using Perseus (version: 1.6.15.0)^2^. The protein (proteome dataset) and peptide isoform table (phospho enriched dataset) were imported. Contaminants, peptide isoforms with no quantitative information, non-unique peptide isoforms, low and medium confident proteins were removed. Only high confident peptide isoforms respectively (master-) proteins were selected. For intra-batch correction of non-normalized peptide isoform (phospho enriched dataset) and protein (proteome dataset) abundances a channel-specific correction factor for each TMT-10plex kit was applied, considering the median of summed intensities per TMT-10plex (plexA(Pat03)-127N-channel was excluded for this study). For inter-batch correction samples were normalized on the control channel of each TMT-10plex (channel 131, pooled sample). Corrected and normalized peptide isoform and protein abundances were Log2-transformed. The matrix was filtered for valid values (100%, in total/all groups) creating a complete matrix. ANOVA-testing was conducted on proteome data at 5% FDR (permutation-based FDR). ANOVA-significant proteins were extracted, abundances were z-scored for hierarchical clustering using ComplexHeatmap R package (version 2.15.4, clustering_distance_rows = "euclidean", clustering_method_rows = "complete", km=4, cluster_columns = FALSE). GO enrichment analysis on clusters was performed (PANTHER version 17.0) using the matrix of ANOVA-significant proteins as background (Fisher’s Exact test, FDR < 1%).  **Isotype specific ELISA**  For exact measurement of IgA1, IgA2 and IgM specific concentrations an ELISA was set up for each isotype with both kappa and lambda detection. To obtain the optimal optical density in the ELISA assay, we determined the optimal coating conditions for all anti-heavy chain capture antibodies. All antibodies were selected based on previous experience or on data provided by the manufacturer. Standard curves ranged from 3.25 to 200 ng/ml, with a linear range between 50 – 200 ng/ml. All antibodies were tested for cross-reactivity. MaxiSorp 96 well plates (NUNC, 442404) were coated overnight with an anti-isotype specific antibody for IgA1 (Southern Biotech, 9130-01), IgA2 (Invitrogen, NBP2-62025) or IgM (Invitrogen Sa5-10291) diluted in PBS. Next, plates were washed three times with 0.05% TWEEN 20 in PBS (PBST) and blocked for 1 hour by incubating with 1% BSA (Roche) in PBST. Standards (IgA1 Bio-Rad HCA189, IgA2 Invivogen #hcd20-mab7, IgM Invitrogen 31146) and ascites samples were diluted in 1% BSA in PBST and incubated for 1.5 hours at room temperature. A mixture of Biotin-labeled-anti-human-kappa and biotin-labeled-anti-human-lambda (Southern biotech, 2062-08 and 2072-08) was used to bind human IgA1, IgA2 or IgM for 1 hour at room temperature. HRP-labeled-streptavidin (Southern biotech 7105-05) was added to bind the biotin labeled antibodies. Plates were washed with three times with PBST after each incubation. Plates were developed with TMB substrate (Invitrogen SB02), reaction was stopped with 1M hydrochloric acid (Fisher chemical 10467640) and read out on Spectramax M3 (Molecular devices) at 450 nm.  **Data and materials availability**  The mass spectrometry proteomics data have been deposited to the ProteomeXchange Consortium via the PRIDE [1] partner repository with the dataset identifier PXD048416. RNAseq reads have been deposited at: DOI 10.5281/zenodo.10797371. Single cell karyotype sequencing data are deposited at EBI-ENA (dataset ID: PRJEB73724). | |
|  |  |

| **Supplementary Figures and Tables** |  |
| --- | --- |

**Figure S1. PIPAC-OX effect on tumor cell karyotypes.**

Single cell shallow sequencing was performed to generate karyotype plots from metastasis samples obtained from (A) patient 3, (B) patient 4, (C) patient 5, (D) patient 9, (E) patient 12, (F) patient 13, and (G) patient 17. Each row represents a single cell.

**A**


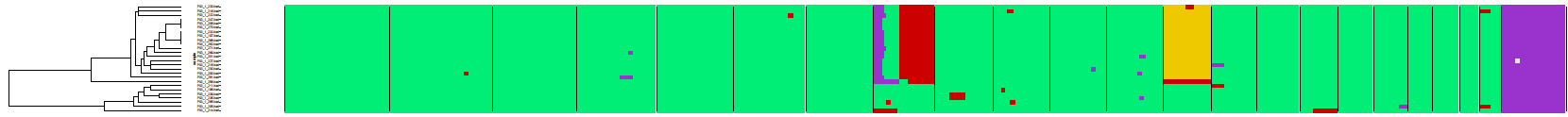

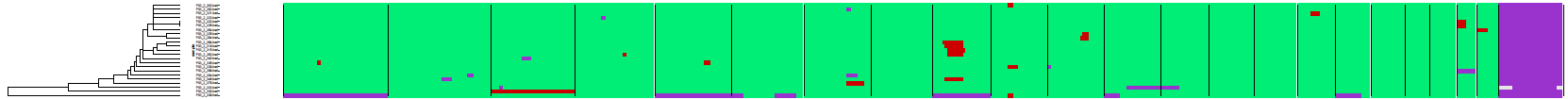


**PIP-03, metastasis 2**

**cycle 1**

**cycle 2**


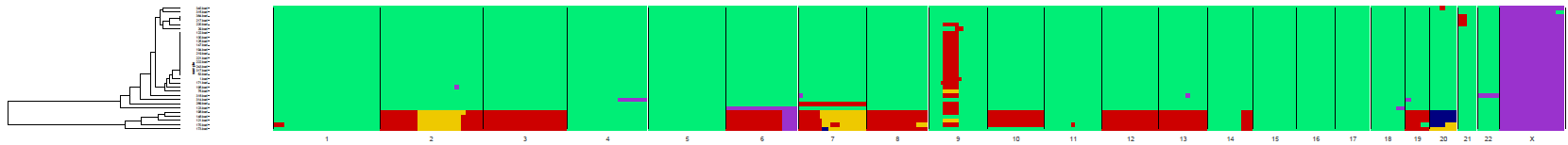


**PIP-04, metastasis 1**

**cycle 1**


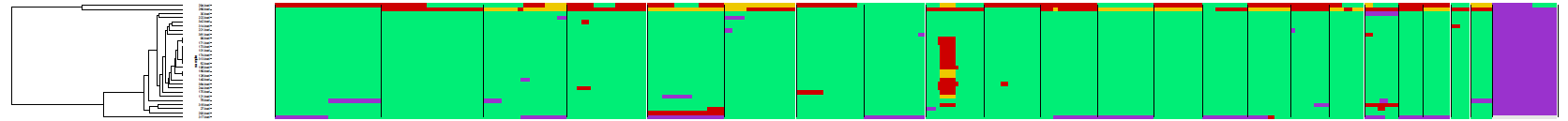


**cycle 3**

**PIP-04, metastasis 2**


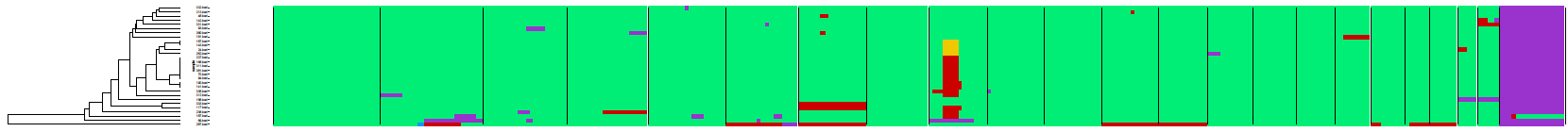


**cycle 3**


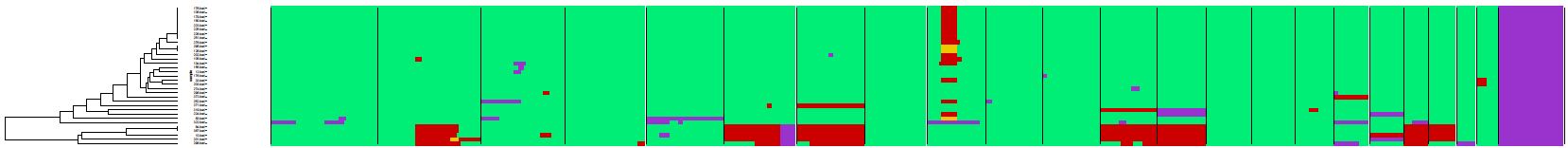


**cycle 1**

**cycle 4**


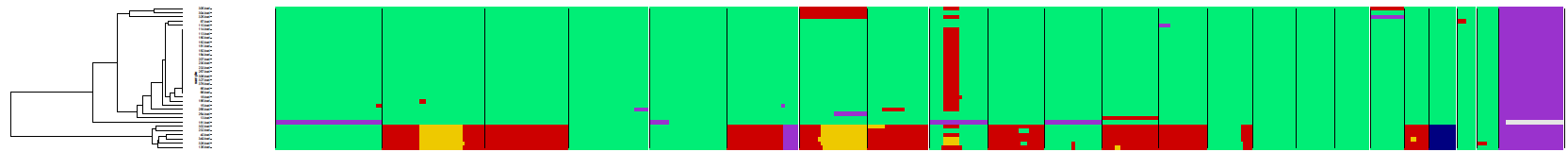


**cycle 4**


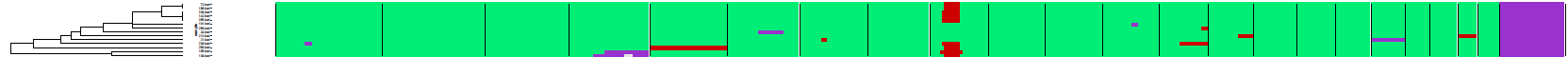

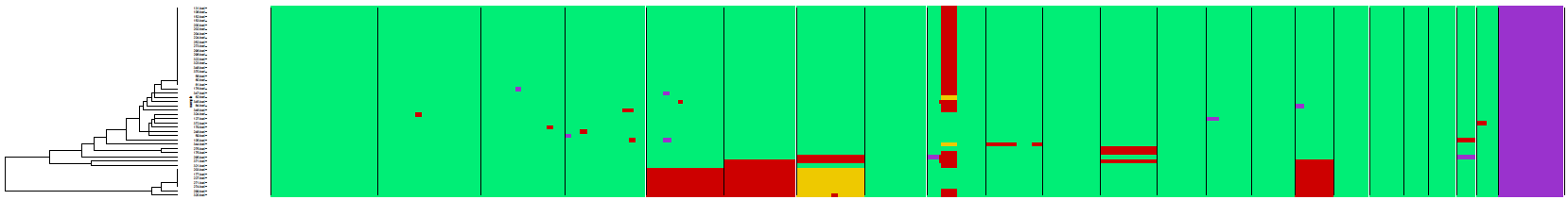

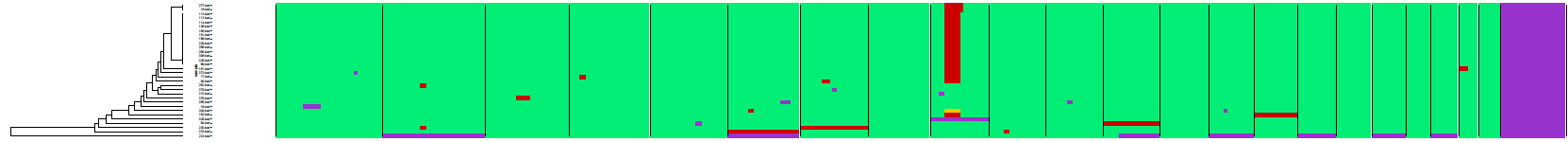


**PIP-04, metastasis 3**

**cycle 3**

**cycle 1**

**B**

**Figure S1. PIPAC-OX effect on tumor cell karyotypes.**

Single cell shallow sequencing was performed to generate karyotype plots from metastasis samples obtained from (A) patient 3, (B) patient 4, (C) patient 5, (D) patient 9, (E) patient 12, (F) patient 13, and (G) patient 17. Each row represents a single cell.

**C**

**PIP-05, metastasis 1**

**cycle 1**

**cycle 3**

**PIP-05, metastasis 2**

**cycle 1**

**cycle 3**

**PIP-05, metastasis 3**


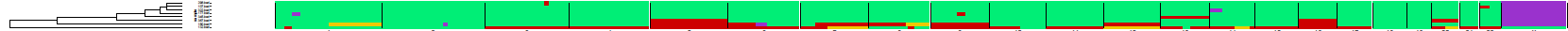

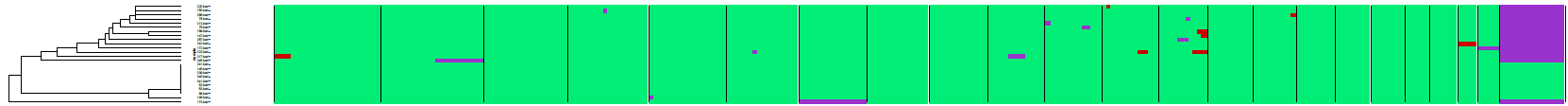

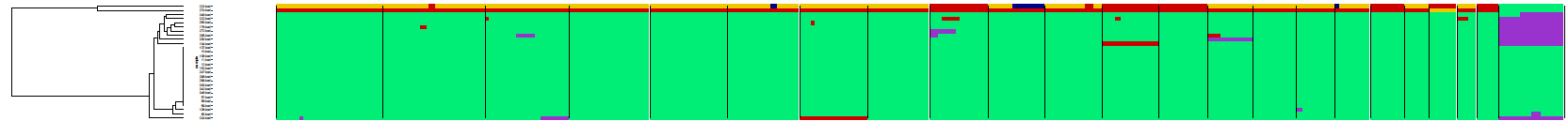

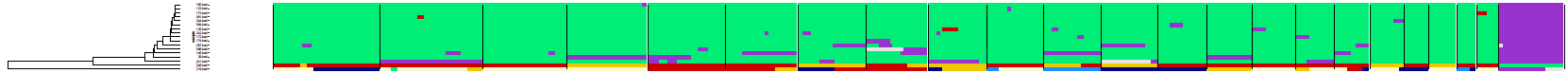


**cycle 1**

**cycle 1**

**PIP-09, metastasis 2**

**D**


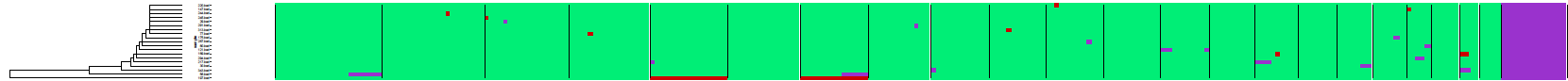

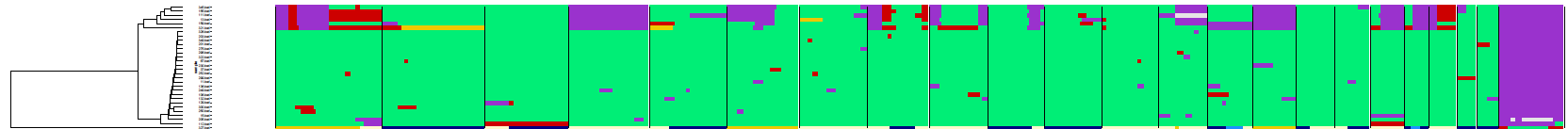


**PIP-09, metastasis 3**

**E**


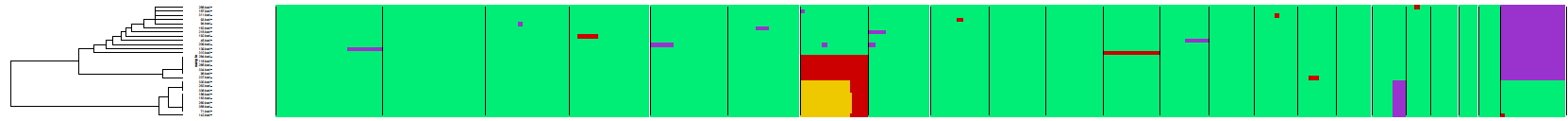


**cycle 1**

**PIP-12, metastasis 3**

**F**

**PIP-13, metastasis 1**

**cycle 2**

**cycle 3**

**PIP-05, metastasis 2**

**cycle 3**

**cycle 2**

**PIP-13, metastasis 2**

**cycle 1**

**cycle 2**

**PIP-13, metastasis 3**

**cycle 3**


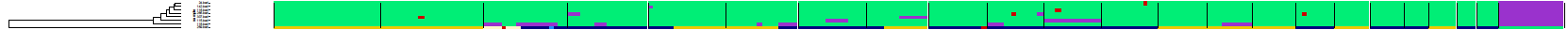

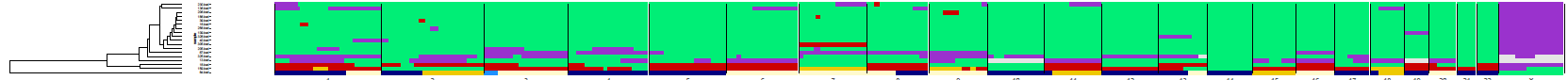

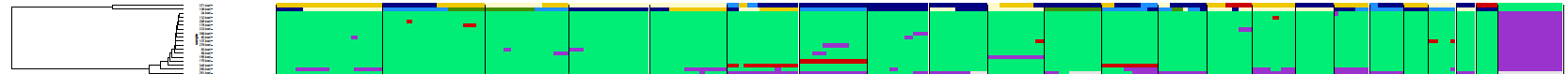

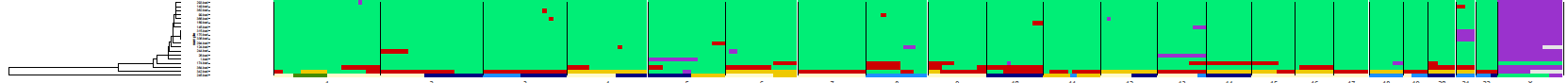

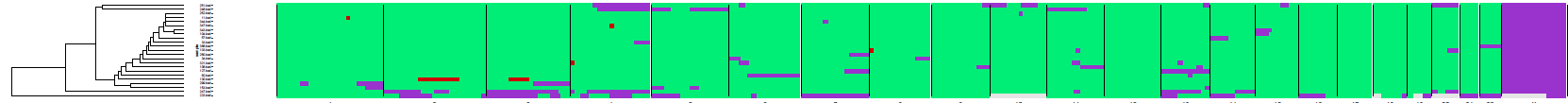

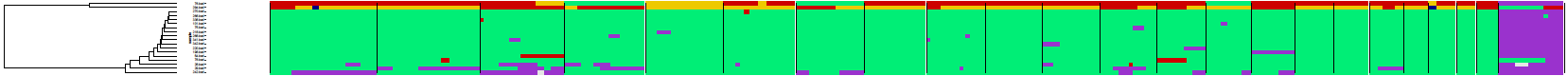

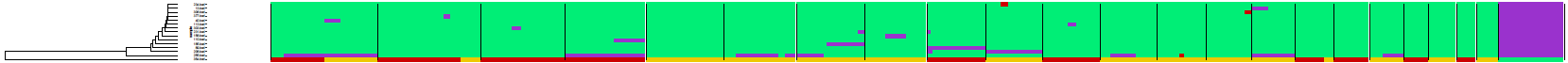


**cycle 2**

**cycle 1**

**G**

**PIP-17, metastasis 1**

**PIP-17, metastasis 2**


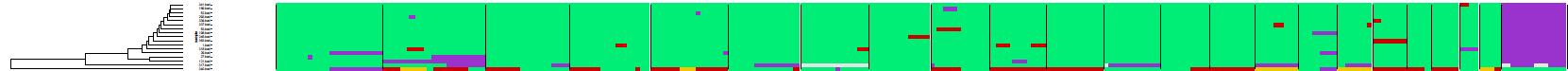

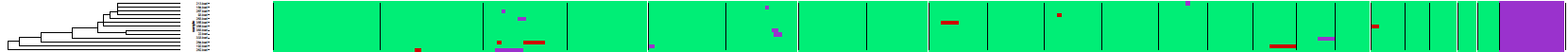

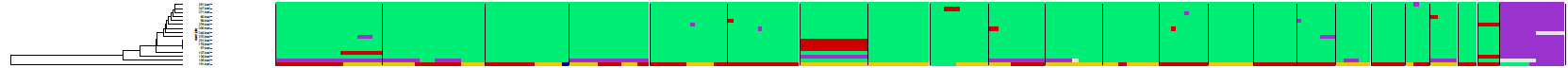

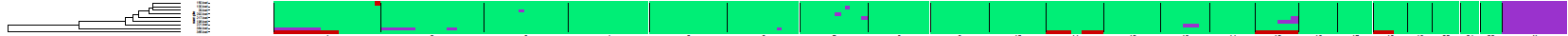

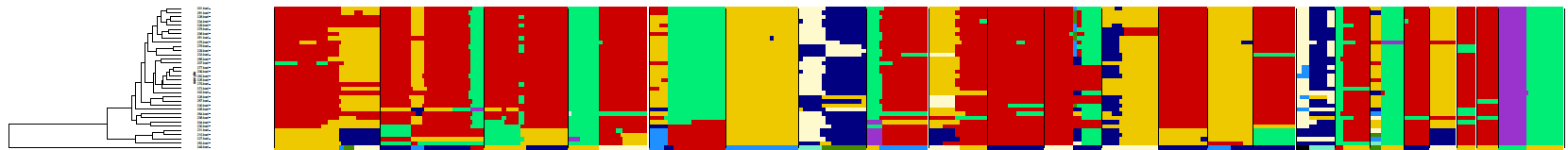

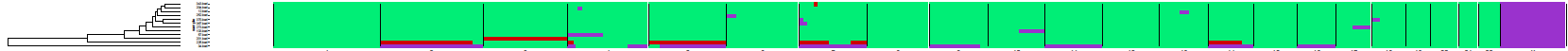


**cycle 2**

**cycle 1**

**cycle 2**

**cycle 1**

**PIP-17, metastasis 3**

**Figure S1. PIPAC-OX effect on tumor cell karyotypes.**

Single cell shallow sequencing was performed to generate karyotype plots from metastasis samples obtained from (A) patient 3, (B) patient 4, (C) patient 5, (D) patient 9, (E) patient 12, (F) patient 13, and (G) patient 17. Each row represents a single cell.

**Figure S2. PIPAC-OX does not influence expression of genesets reflecting cell proliferation or apoptosis.**

Dotplots showing expression of the following gene sets in treatment-naïve *versus* treated peritoneal metastasis samples: (A) KEGG ‘cell cycle’, (B) Gene Ontology ‘cell division’, (C) KEGG ‘apoptosis’, (D) Gene Ontology ‘apoptotic signaling pathway’. (E) Immunohistochemistry using Ki67 on sets in treatment-naïve *versus* treated peritoneal metastasis samples. (F) Representative images from (E).

p=0.4289

p=0.589

**A**

**B**

**C**

**D**

p=0.663

p=0.349

**E**


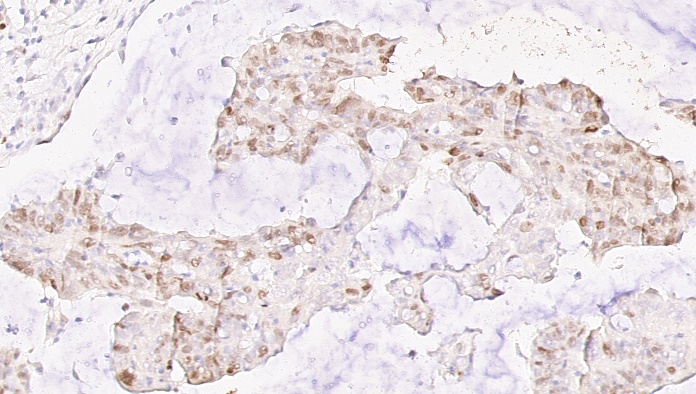

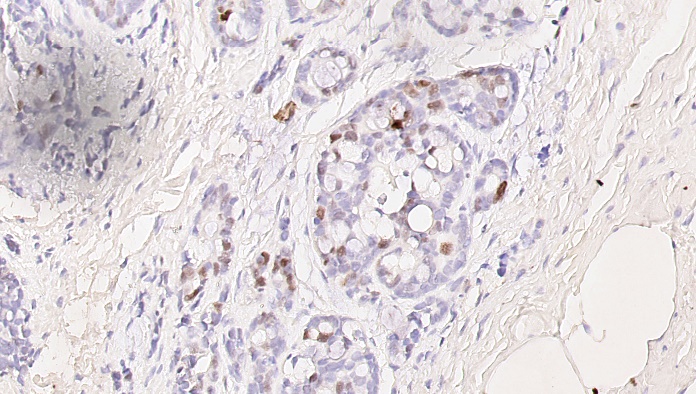

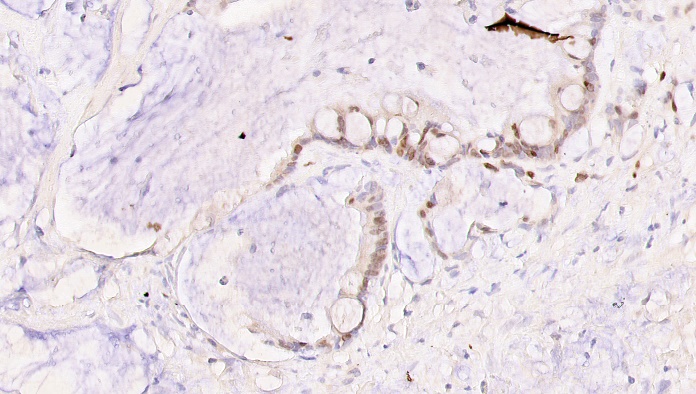

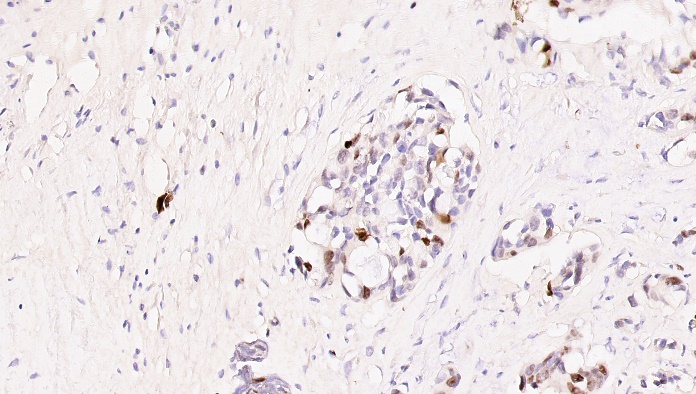

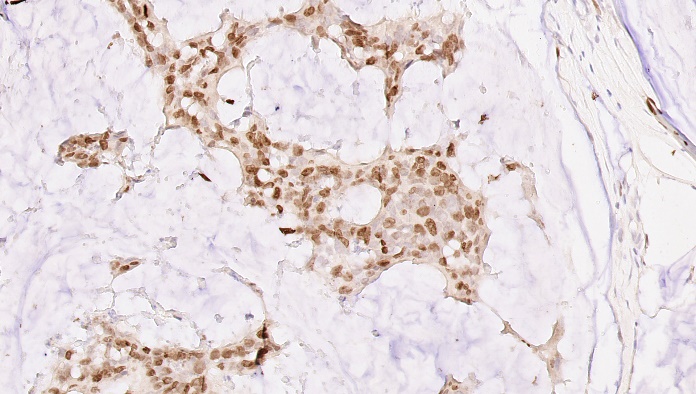

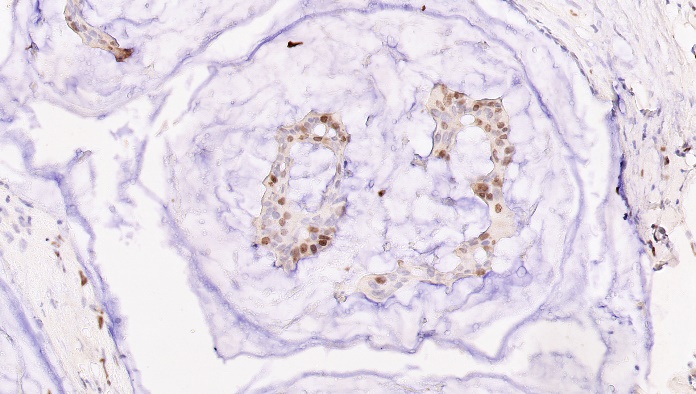


**F**

cycle 1

cycle 2

cycle 3

p=0.5508

**Figure S3. Reduced expression of genes reflecting hypoxia following ePIPAC is paralleled by increased expression of B and T cell signatures.**

Dotplots showing expression of (A) HIF1A target genes (ref. 12), (B) CA9, (C) B cells (ref 14) (D) plasma cells (ref 16) (E) CD8+ T cells (ref 14), and (F) cytotoxic T lymphocytes (CTL) (ref 15) over time. Biopsies were obtained just prior to each treatment cycle. Cycle 1 samples therefore are therefore PIPAC-naïve.

**A**

**B**

**C**

**D**

**E**

**F**

**Figure S4 Metastasis-associated TLS contain MECA79^+^ high endothelial vanules (HEV).**

Immunohistochemistry using anti-MECA79 on treatment naive (cycle 1) and ePIPAC-treated metastases. MECA79-positive venules are detected within the immune cell aggregates induced by ePIPAC.


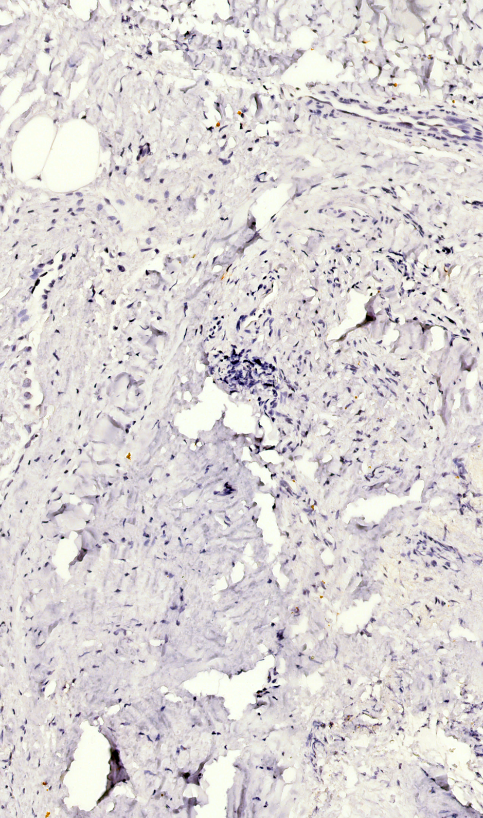

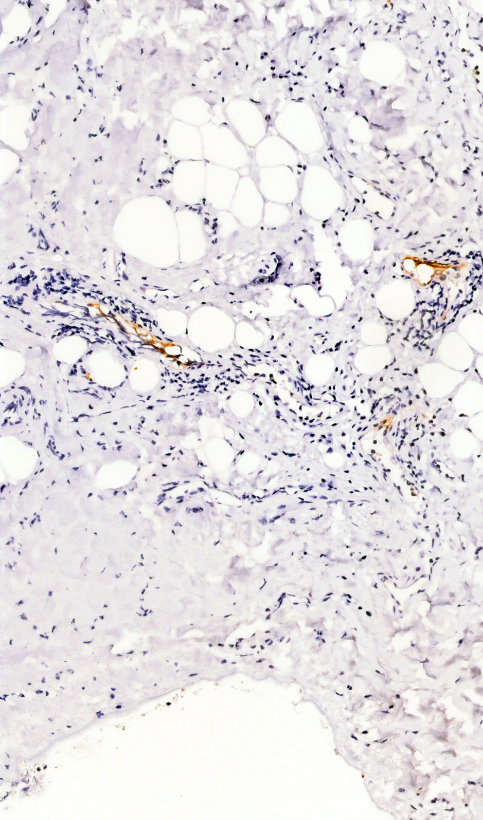

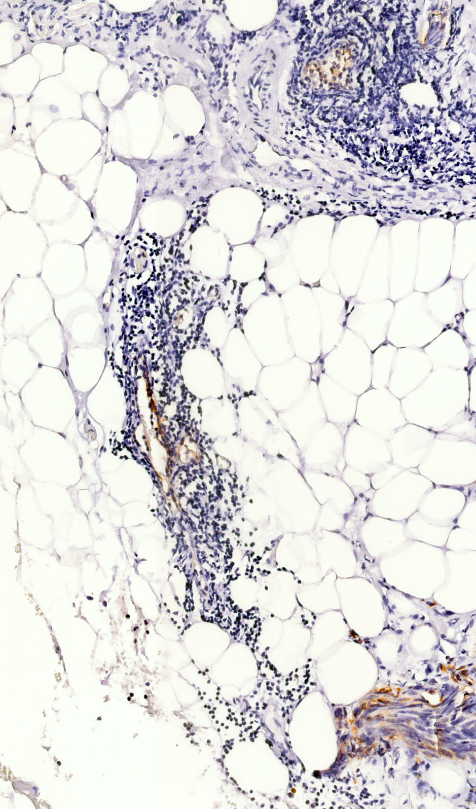


cycle 1

cycle 2

cycle 3


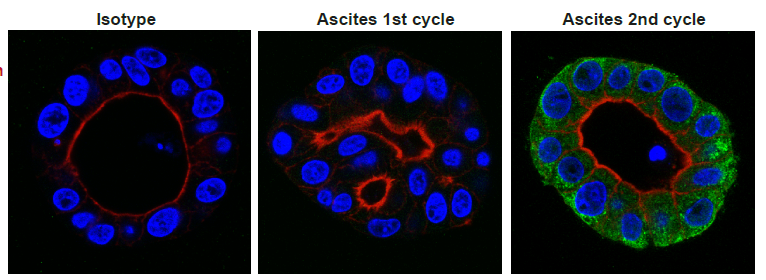


anti-human IgG; phalloidin; DAPI

isotype

ascites – cycle 1

ascites – cycle 2

**C**

**B**

stronger recognition

by ascites-cycle 2

stronger recognition

by ascites-cycle 1

**A**


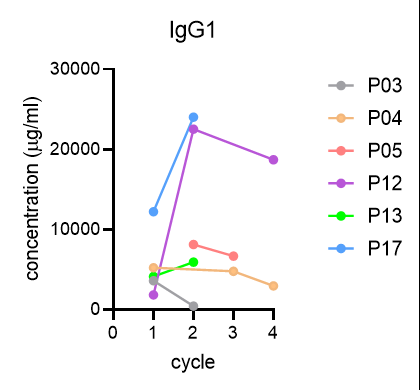


IgG1

IgG3

IgG4

IgA1

IgA2

IgM

IgG2

**FIGURE S5. PIPAC triggers the generation of tumor-reactive antibodies.**

(A) Enzyme-linked immunosorbent assay (ELISA) quantification of the concentrations (in mg/ml) of the indicated immunoglobulins in ascites samples collected prior to PIPAC (cycle 1) and following treatment (cycle 2-4). Individual patients are color-coded. The grey bars indicate the concentration range of each immunoglobulin found in human plasma. (B) Human proteome array analysis of the reactivity of antibodies in ascites samples from patient 12, prior to and following PIPAC. The plot shows ratios of the intensity of antigen recognition by antibodies from both samples. Positive values indicate stronger recognition by antibodies from the post-treatment sample. Negative values indicate stronger recognition by antibodies from the pre-treatment sample. Each dot represents an individual antigen (23.133 in total). The identities of the differentially recognized antigens are ranked and listed in Table S8. (C) Fixed patient-derived organoids (patient 3) were stained with human IgG1 (control) or ascites samples obtained prior to and following PIPAC. Antibody reactivity was then analyzed by immunofluorescence and subsequent confocal microcopy.

**Table S1. The PIPAC study biobank.**

**Table S2.** Differential gene expression analysis of Hallamrk and hypoxia signatures between treatment-naïve and PIPAC-treated samples.

**Table S3.** Differential gene expression analysis of immune cell signatures between treatment-naïve and PIPAC-treated samples.

**Table S4.** PIPAC-indcued changes in protein abundance per cluster. Data available on request. (table too large for this Supplemental file).

**Table S5.** Gene Ontology terms enriched in cluster 2.

**Table S6.** Immunoglobulins in cluster 2

**Table S7.** Phospho-proteomics analysis of PIPAC-induced STAT phosphorylation. Data availbale on request. (Table too large for this Supplemental file).

**Table S8.** PIPAC-induced changes in the expression of genes involved in immunosuppression (RNA)

**Table S9.** Spot intensities on the HuProt array. Data available on request (table too large for this Supplemental file).

References

1. Taus T, Kocher T, Pichler P, Paschke C, Schmidt A, Henrich C, Mechtler K. Universal and confident phosphorylation site localization using phosphoRS. *J Proteome Res* 2011;**10**(12): 5354-5362.

2. Tyanova S, Temu T, Sinitcyn P, Carlson A, Hein MY, Geiger T, Mann M, Cox J. The Perseus computational platform for comprehensive analysis of (prote)omics data. *Nature methods* 2016;**13**(9): 731-740.
